# Proteomic analysis of mitochondria associated membranes in renal ischemic reperfusion injury

**DOI:** 10.1186/s12967-024-05021-0

**Published:** 2024-03-10

**Authors:** Yi Li, Hua-bin Wang, Jin-long Cao, Wen-jun Zhang, Hai-long Wang, Chang-hong Xu, Kun-peng Li, Yi Liu, Ji-rong Wang, Hua-lan Ha, Sheng-jun Fu, Li Yang

**Affiliations:** 1 Department of Urology, Institute of Urology, Gansu Urological Clinical Center, Lanzhou University Second Hospital, Lanzhou, 730030 Gansu China; 2 Department of Anesthesiology, Lanzhou University Second Hospital, Lanzhou, 730030 Gansu China; 3 Department of Nephrology, Lanzhou University Second Hospital, Lanzhou, 730030 Gansu China; 4 Department of Nephrology, The First People’s Hospital of Lanzhou City, Lanzhou, 730030 Gansu China

**Keywords:** Proteomics analysis, Mass spectrometry, Mitochondria associated membranes, Kidney, Ischemic reperfusion injury

## Abstract

**Background:**

The mitochondria and endoplasmic reticulum (ER) communicate via contact sites known as mitochondria associated membranes (MAMs). Many important cellular functions such as bioenergetics, mitophagy, apoptosis, and calcium signaling are regulated by MAMs, which are thought to be closely related to ischemic reperfusion injury (IRI). However, there exists a gap in systematic proteomic research addressing the relationship between these cellular processes.

**Methods:**

A 4D label free mass spectrometry-based proteomic analysis of mitochondria associated membranes (MAMs) from the human renal proximal tubular epithelial cell line (HK-2 cells) was conducted under both normal (N) and hypoxia/reperfusion (HR) conditions. Subsequent differential proteins analysis aimed to characterize disease-relevant signaling molecules. Gene Ontology (GO) and Kyoto Encyclopedia of Genes and Genomes (KEGG) analysis was applied to total proteins and differentially expressed proteins, encompassing Biological Process (BP), Cell Component (CC), Molecular Function (MF), and KEGG pathways. Further, Protein–Protein Interaction Network (PPI) exploration was carried out, leading to the identification of hub genes from differentially expressed proteins. Notably, Mitofusion 2 (MFN2) and BCL2/Adenovirus E1B 19-kDa interacting protein 3(BNIP3) were identified and subsequently validated both in vitro and in vivo. Finally, the impact of MFN2 on MAMs during hypoxia/reoxygenation was explored through regulation of gene expression. Subsequently, a comparative proteomics analysis was conducted between OE-MFN2 and normal HK-2 cells, providing further insights into the underlying mechanisms.

**Results:**

A total of 4489 proteins were identified, with 3531 successfully quantified. GO/KEGG analysis revealed that MAM proteins were primarily associated with mitochondrial function and energy metabolism. Differential analysis between the two groups showed that 688 proteins in HR HK-2 cells exhibited significant changes in expression level with P-value < 0.05 and HR/N > 1.5 or HR/N < 0.66 set as the threshold criteria. Enrichment analysis of differentially expressed proteins unveiled biological processes such as mRNA splicing, apoptosis regulation, and cell division, while molecular functions were predominantly associated with energy metabolic activity. These proteins play key roles in the cellular responses during HR, offering insights into the IRI mechanisms and potential therapeutic targets. The validation of hub genes MFN2 and BNIP3 both in vitro and vivo was consistent with the proteomic findings. MFN2 demonstrated a protective role in maintaining the integrity of mitochondria associated membranes (MAMs) and mitigating mitochondrial damage following hypoxia/reoxygenation injury, this protective effect may be associated with the activation of the PI3K/AKT pathway.

**Conclusions:**

The proteins located in mitochondria associated membranes (MAMs) are implicated in crucial roles during renal ischemic reperfusion injury (IRI), with MFN2 playing a pivotal regulatory role in this context.

**Supplementary Information:**

The online version contains supplementary material available at 10.1186/s12967-024-05021-0.

## Introduction

An acute kidney injury (AKI) commonly occurs as a result of ischemic reperfusion, which refers to the damage of renal tissue caused by ischemia followed by reperfusion. This phenomenon can lead to further aggravation of injury rather than reduction. Various factors such as hypotension after shock, left heart function failure, cardiopulmonary resuscitation (CPR), renal transplantation, clinical disease and surgery can cause varying degrees of renal ischemic reperfusion injury (IRI). This is particularly true for kidney transplantation, in which the ischemic reperfusion process is an inevitable step. Prolonged ischemia increases the incidence of early and late allograft renal dysfunction [1]. The loss of tubular epithelial cell (TEC) function caused by IRI can result in the development of acute kidney injury, delayed graft function, and acute and chronic organ rejection [2]. Therefore, it is a significant clinical challenge that clinicians must face during kidney transplant surgery to prevent renal IRI.

The mechanism of kidney IRI mainly involves a reduction in ATP, mitochondrial dysfunction, intracellular Ca^2+^ accumulation, excessive reactive oxygen species (ROS), release of proinflammatory cytokines, and cell apoptosis, et al. [3–8]. During the ischemic phase, oxygen and nutrient supply to the renal tissue are disrupted, leading to reduced ATP levels and impaired cellular metabolism. Upon reperfusion, a sudden influx of oxygen and nutrients causes an abrupt increase in oxidative stress and ROS production, which can cause cellular damage and contribute to the development of inflammation and apoptosis. In addition to ROS generation, the accumulation of intracellular calcium ions is also a major contributor to the pathogenesis of renal IRI. The influx of calcium ions during the reperfusion phase can activate a variety of calcium-dependent enzymes, which can lead to cellular damage and apoptosis. Therefore, renal ischemic reperfusion injury is a common cause that can lead to serious renal dysfunction. Given the imperative need for a comprehensive comprehension of the mechanisms behind kidney ischemic reperfusion injury (IRI) to formulate effective therapeutic strategies, the investigation into mitochondria associated membranes (MAMs) emerges as a pivotal avenue for deeper insights. While specific clinical therapies directly targeting mitochondrial control or MAMs are currently lacking, the future outlook for MAMs research involves delving deeper into their roles in disease mechanisms. The objective is to translate this knowledge into more effective treatment strategies, ultimately improving clinical outcomes for patients.

Mitochondria and the endoplasmic reticulum (ER) are crucial organelles in eukaryotic cells, each serving distinct biological functions. Mitochondria function as the cell's powerhouse, generating ATP through oxidative phosphorylation and playing roles in apoptosis, calcium ion regulation, and cellular metabolism. ER constitutes a complex membrane system responsible for protein synthesis and folding, calcium ion storage, lipid synthesis regulation, among other functions. These two organelles establish physical and biochemical interactions at specific subdomains described as mitochondria associated endoplasmic reticulum membranes (MAMs), which are not formed through membrane fusions but rather utilize proteinaceous tethers. These tethers facilitate direct, rapid, and bidirectional transport of signaling molecules between the mitochondria and ER, characterizing the structural and functional relationship between these two organelles. MAMs play pivotal roles in various biological processes, including modulation of lipid metabolism [9], regulation of mitochondrial dynamics [10], the initiation of mitophagy [11–13], control of redox signaling [14, 15], ER stress responses [16], calcium homeostasis [17–19], the assembly of inflammasomes [20] and apoptosis [21]. MAMs cover about 4–20% of the mitochondrial surface, depending on the stress and metabolic state of their cells [18]. In different physiological and pathological states, the number and morphology of MAMs and the distance between mitochondria and ER may vary.

While the existence of the mitochondria associated membranes (MAMs) was initially observed more than five decades ago [22], comprehensive exploration of MAMs began in earnest only after the development of a dependable isolation technique, which was introduced by Vance in 1990 [23]. Subsequently, more and more researches had been carried on MAMs and many key breakthroughs were provided for understanding MAMs function. For example, a growing body of evidence suggests MAMs play pivotal roles in the onset and progression of various conditions, including neurological diseases [24], cardiovascular diseases [25], renal disorders [26] and so on. These findings propel the field of MAMs research forward, fostering a more nuanced understanding of their functional implications and potential clinical applications.

The MAMs contains multitudinous chaperones, oxidoreductases. As mentioned above, this subcellular compartment likely plays a role in various cellular metabolic processes by coordinating tasks such as protein folding, lipid synthesis, calcium regulation, and redox reactions. Disruptions in MAMs function can lead to reduced ATP production in mitochondria, increased generation of reactive oxygen species (ROS), heightened ER stress, calcium overload, and ultimately, apoptosis. When considering the mechanisms underlying the onset and progression of ischemia reperfusion injury (IRI), the findings regarding MAMs suggest a potential involvement of MAMs in the development of renal IRI. However, few studies have examined the influences of renal IRI on the structure, signaling and functions of MAMs. To gain insight into how IRI affects MAMs in kidney, we conducted quantitative proteomics of mitochondria associated membranes using 4D label free MS analysis in a model of hypoxia/reoxygenation of human renal proximal tubular epithelial cell line (HK-2 cells), which simulated the process of renal ischemic reperfusion. This is the first reported quantitative proteomic analyses of this suborganelle in renal IRI. Our data provided a map of HK-2 cells proteomic changes to understanding the mechanism of acute kidney injury (AKI) caused by IRI and these data provide us with a possibility of defining the relationship between MAM proteins and AKI. Quantitative validation was also conducted for a few MAM proteins and structure changes between normal and HR HK-2 cells.

## Materials and methods

### Hypoxia/reoxygenation of HK-2 cells

HK-2 cells (a human renal epithelial cell line with characteristics of proximal tubular cells, purchased from shanghai fu heng cell, catalog number FH0228) were incubated with a complete Dulbecco's Modified Eagle Medium (DMEM/F12) (Gibco, USA) containing 10% fetal bovine serum (FBS) (PAN,Germany) and 1% Penicillin/Streptomycin mixture in an incubator (Thermo Fisher Scientific, USA) at 37 °C, 21% O_2_, and 5% CO_2_. The cells hypoxia/reoxygenation (HR) model was developed by exposing HK-2 cells to hypoxic conditions (37 °C, 1% O_2_, 94% N_2_, and 5% CO_2_) for 8 h in glucose- and serum-free medium. Subsequently, HK-2 cells were reoxygenated for 2 h by incubating in a fresh complete DMEM/F12 under the normal condition. 5% CO_2_ and 21% O_2_ were the culture conditions for the normal group. The hypoxia/reoxygenation model was based on established methodologies from prior research [27], as well as informed by preliminary experiments conducted in our laboratory.

### Isolation of MAMs from HK-2 cells

We compared 6 individual cell samples for proteomic analysis between the HR group and the control group (n = 3 for 3 each group, and each sample contained 1–2*10^8^ cells).The MAMs isolation followed a previously described protocol [28] with slight modifications (see Additional file 1: Fig. S1). As follows: completing the homogenization process when 70–80% of cells damage has been achieved, rather than 80–90%, aiming to minimize HK-2 cells mitochondrial injury. Additionally, we have transitioned to a 38.5 ml polyallomer ultracentrifuge tubes instead of a 15 ml percoll system to meet the required proteinomics quantities. Briefly, 1–2*10^8^ HK-2 cells were combined and manually homogenized on ice using a glass homogenizer (Solarbio, China) with 60–80 strokes. Two centrifugations at 600*g* were used to remove unbroken cells and nuclei from the homogenate. Further centrifugation at 7000*g* for 5 min obtained crude mitochondria from the supernatant. We resuspended the crude mitochondrial fractions in mitochondrial resuspension buffer (MRB, containing 250 mM mannitol, 5 mM HEPES and 0.5 mM EGTA). This suspension was gently layered on top of a Percoll medium (containing 225 mM mannitol, 25 mM HEPES, pH 7.4, 1 mM EGTA, and 30% Percoll, v/v). Following centrifugation at 95,000*g* for 30 min, the crude MAMs fraction appeared as a diffuse white band above, while a dense band containing purified mitochondria settled near the bottom of the tube. Subsequently, the MAMs fraction was gathered and washed to eliminate the Percoll. After centrifugation at 6300*g* for 10 min, the supernatant was centrifuged at 100,000*g* for further purification. All fractions obtained were rapidly frozen using liquid nitrogen and stored at − 80 °C until they were ready to be used.

### Proteolytic digestion

To prepare for digestion, the protein solution was first subjected to reduction by 5 mM dithiothreitol for 30 min at 56 °C, followed by alkylation using 11 mM iodoacetamide for 15 min at room temperature in darkness. Subsequently, the protein sample was diluted by the addition of 100 mM TEAB until the urea concentration reached below 2 M. Then, trypsin (Progema, China) was added at 1:50 trypsin-to-protein mass ratio for the first digestion overnight and 1:100 trypsin-to-protein mass ratio for a second 4 h-digestion at 37 °C. Finally, the peptides were desalted by C18 SPE column.

### 4D mass spectrometer

A reversed-phase analytical column (25 cm in length, 75/100 mm in diameter) was directly loaded with the tryptic peptides dissolved in solvent A (0.1% formic acid, 2% acetonitrile). The peptides were separated using a gradient as follows: 6% to 24% solvent B (0.1% formic acid in acetonitrile) over 70 min, 24% to 35% in 14 min, followed by a rapid increase to 80% in 3 min, and then held at 80% for the final 3 min. This separation was performed at a constant flow rate of 450 nL/min on a nanoElute UHPLC system (Bruker Daltonics). The peptides were analyzed using capillary source and timsTOF Pro mass spectrometry (Bruker Daltonics). This state-of-the-art mass spectrometer is equipped with advanced features, including trapped ion mobility spectrometry (TIMS), which enhances the separation and resolution of ions. An electrospray voltage of 1.60 kV was applied, and both precursors and fragments were detected by the TOF detector, covering a MS/MS scan range from 100 to 1700 m/z. The timsTOF Pro operated in parallel accumulation serial fragmentation (PASEF) mode. It selected precursors with charge states from 0 to 5 for fragmentation, acquiring 10 PASEF-MS/MS scans in each cycle. Dynamic exclusion was set to 30 s.

### Protein identification and quantification

The MS/MS data were analyzed using MaxQuant search engine (v.1.6.15.0). Tandem mass spectra were searched against the human SwissProt database (20422 entries) combined with a reversed decoy database. Trypsin/P was chosen as the cleavage enzyme, allowing a maximum of 2 missed cleavages. Precursor ion mass tolerance was set at 20 ppm for the initial search and 5 ppm for the main search, while fragment ion mass tolerance was set at 0.02 Da. Fixed modifications included carbamidomethyl on Cys, and variable modifications included acetylation on protein N-terminal and oxidation on Met. The false discovery rate (FDR) was adjusted to be less than 1%. Quantification was performed using a 4D label-free quantification method.

### Development of a rat IRI model in vivo

Male Wistar rats, weighing 200–220 g, were sourced from the Lanzhou Veterinary Research Institute of the Chinese Academy of Agricultural Sciences. These rats received sterile food and unrestricted access to water. They were divided into two groups: a sham group (N, n = 6) and an ischemic reperfusion group (IR, n = 6).

Rats in the IR group underwent a midline abdominal incision. Ischemia was induced by clamping the bilateral renal pedicles with arterial clips for 40 min, followed by releasing the arterial clips to initiate reperfusion for 2 h. The Sham group only underwent an incision without experiencing ischemic reperfusion injury (IRI).

Kidneys were promptly harvested from both the Sham and IR groups. Some kidney samples were fixed with 4% paraformaldehyde (P1110, Solarbio, China), while the remaining samples were stored at − 80 °C for subsequent analyses. All experiments adhered to the Law on Laboratory Animals and Care and received approval from the Ethics Committee of the Second Hospital of Lanzhou University, China.

### RNA isolation and quantitative real-time polymerase chain reaction (qRT-PCR)

Total RNA was extracted from cell lines using Trizol reagent (Solarbio, China). First-strand cDNA was synthesized using the Evo M-MLVRT Kit (Accurate Biotechnology, China) with 2 μg of total RNA following the manufacturer’s instructions. qRT-PCR was performed on a CFX96 real-time PCR detection system (Bio-Rad, Hercules, CA, USA), and a SYBR Green Premix Pro Taq HS qPCR Kit (Accurate Biotechnology, China) was used for gene detection. Actin was used as an internal control to calculate the relative mRNA levels. Primers for MFN2 were as follows: forward, 5′-GCAGAAGGCTTTCAAGTGAGGAT-3′; reverse, 5′-GGTCTTGCCGCTCTTCACG-3′. Primers for BNIP3 were as follows: forward, 5′-CAGCAATAATGGGAACGGGG-3′; reverse, 5′-GAGCGAGGTGGGCTGTCA-3′. qRT-PCR was performed according to the manufacturer’s instructions. Briefly, the cycling reaction was as follows: initial denaturation at 95 °C for 30 s, followed by 50 cycles of denaturation at 95 °C for 10 s and annealing at 60 °C for 30 s.

### Western blotting

For validation, we employed the identical samples that had been utilized in the mass spectrometry analysis. BCA protein assays were used to determine protein concentration. For each sample, proteins of 30 μg were electrophoresed with 12.5% sodium dodecyl sulfate–polyacrylamide gel electrophoresis (SDS-PAGE) and transferred to PVDF membranes. Membranes were blocked with 5% TBST containing skimmed milk powder for 1 h at room temperature, and incubated with primary antibodies overnight at 4 °C. The following primary antibodies were used: β-actin (1:2000,20536–1-AP, Proteintech, China), MFN2(1:2000,12186-1-AP, Proteintech, China), GRP75 (1:2000,14887-1-AP, Proteintech, China),VDAC1(1:2000,155259-1-AP, Proteintech, China), IP3R(1:500,19962-1-AP, Proteintech, China), CytC (1:2000,10993-1-AP, Proteintech, China), TOM20 (1:3000,11802-1-AP, Proteintech, China), COXIV(1:6000,11242-1-AP, Proteintech, China), BNIP3(1:1000,WL01139,Wanleibio, China), PI3K (1:500,WK02240,Proteintech, China), p-PI3K(1:1000,#AF3242, Affinity, China),AKT(1:5000,10176-2-AP,Proteintech, China), p-AKT(1:5000,66444-1-Ig,Proteintech, China). After incubation with antibody diluent containing secondary antibody for 1 h at room temperature, membranes were scanned on ECL detection system (chemiluminescence). Data from densitometry of protein band intensities was analyzed with ImageJ.

### Renal histology and immunohistochemistry (IHC)

The kidney samples, embedded in paraffin, were uniformly sectioned at a thickness of 4 μm. Subsequently, sections underwent a systematic process of deparaffinization and hydration, followed by staining with hematoxylin and eosin (H & E) as well as immunohistochemical (IHC) studies. Morphological assessments were observed by two experienced renal pathologists who were blinded to the treatments. The histopathological score of kidney injury was determined according to the acute tubular necrosis scoring method. Ten high-power fields were randomly selected from each slide at the corticomedullary junction, according to the degree of injury, as follows: < 5%, 5–25%, 25–50%, 50–75%, and > 75%, which were rated as 0, 1, 2, 3, and 4 points, respectively.

For the IHC analysis, the kidney tissue sections were incubated with specific antibodies as follows: MFN2 polyclonal antibody (1:200,12186-1-AP, Proteintech, China) and BNIP3 monoclonal antibody (1:200,68091-1-Ig,Protintech,China). After an overnight incubation at 4 °C, the samples were incubated with secondary antibodies for 1 ha t 22 °C, treated with diaminobenzidine tetrahydrochloride (DAB), and counterstained with haematoxylin. The sections were observed by light microscopy (Olympus, Japan).

### Immunofluorescence (IF) staining

Paraffin-embedded samples were also utilized for immunofluorescence (IF) studies. Antigen retrieval was conducted using a 0.01 mol/L citrate buffer (pH 6.0) in a pressure cooker for 3 min. To minimize non-specific binding, QuickBlock™ Blocking Buffer for Immunol Staining was applied and incubated for 30 min at room temperature. Following this, the samples were treated with specific primary antibodies overnight at 4 °C and subsequently exposed to secondary antibodies conjugated with Fluor or FITC (Affinity, China) for 1 h at room temperature. The sections were then sealed with Antifade Mounting Medium for Fluorescence (with DAPI) (Biosharp, China).

Primary antibodies utilized for IF staining included MFN2 (1:200, Protintech, China) and BNIP3 (1:200, Protintech, China). Immunofluorescence images were captured using an upright fluorescence microscope (BX53 + DP74, Olympus, Japan).

### Lentivirus infection

Serving as a hub gene and a crucial regulator of mitochondrial dynamics and MAM integrity, the overexpression of MFN2 was employed to provide a focused avenue for dissecting the intricate interplay between MFN2 and MAMs within the context of our study.

HK-2 cells were transfected with either MFN2 overexpression lentivirus or GFP control lentivirus (Hanheng Company, Shanghai, China, MOI = 4) for 48 h. Then, the DMEM/F-12 containing 5.0 μg/μl puromycin was replaced for screening puromycin-resistant clones to finally obtain stable MFN2 overexpression cell lines.

### Transfection of SiRNA

To validate our findings from an orthogonal perspective, we employed small interfering RNA (siRNA) technology to knock down MFN2, offering a multi-faceted approach to verification. The small interfering RNA (siRNA) oligonucleotides targeting MFN2 were commercially synthesized by Gene Pharma (Shanghai, China) and introduced into cells through transfection utilizing the GP (GenePharm, Shanghai, China) transfer transfection reagent. The siRNA sequences are as follows: siMFN2-1 (sense: 5′-GGCCAAACAUCUUCAUCCUTT-3′, antisense: 5′-AGGAUGAAGAUGUUUGGCCTT-3′); siMFN2-2 (sense: 5′-GCUCUUGGCUCAAGACUAUTT-3′, antisense: 5′-AUAGUCUUGAGCCAAGAGCTT-3′); siMFN2-3 (sense: 5′-CCCUCAACUAUGACCUAAATT-3′, antisense: 5'-UUUAGGUCAUAGUUGAGGGTT-3'). The effectiveness of MFN2 silencing was assessed via western blotting after 48 h.

### Confocal microscopy

MAMs was visualized using fluorescent probes that selectively labeled mitochondria (MitoTracker in red, Beyotime, China) and the ER (ER Tracker in green, Thermo Fisher Scientific, USA) in live cells by confocal microscopy (Zeiss LSM 510 microscope, Germany)with a 60 × 1.3 NA oil-immersion objective. Mitochondrial fluorophores were excited using a 633 nm laser, and fluorescence emissions were recorded at 558–617 nm. Similarly, ER fluorophores were excited with a 504 nm laser, and their fluorescence emissions were recorded at 511 nm. Manders' colocalization coefficient [29] was employed to quantify the interaction between mitochondria and ER using the Fiji software.

### Transmission electron microscopy (TEM)

HK-2 cells (5*10^6^ for N and HR group, respective) were fixed at 4 °C with 0.5% glutaraldehyde in a 0.01 mol/L phosphate buffer saline for 10 min before centrifuged with 10,000 for 10 min, and then sediment was postfixed with 3% glutaraldehyde at 4 °C. Tissue were prefixed with 3% glutaraldehyde and refixed with 1% osmium tetroxide. Approximately 60–90 nm ultrathin sections were mounted on cooper meshes. A JEM-140 0FLASH transmission electron microscope (Japan) was employed to collect images of copper meshes, with each individual copper mesh undergoing observation at magnifications of up to 6000 times. The percentage of mitochondria membrane in contact with ER within a 50 nm range was measured and normalized to mitochondria perimeter, as previously reported [30].

### Bioinformatics analysis

All bioinformatics analyses were conducted using R v.4.3.2 software. We performed data normalization using the LIMMA R package's normalizeBetweenArrays function for the sequencing data. Differential analysis was conducted with P-value < 0.05 and N/HR > 1.5 or N/HR < 0.66 as a cut-off value. Subcellular localization predictions were made using the WolfPSORT tool. Enrichment analysis was conducted using the Database for Annotation, Visualization, and Integrated Discovery (DAVID) website (https://david.ncifcrf.gov/). The proteins were categorized based on Gene Ontology annotations in biological processes, cellular components, and molecular functions. Additionally, the analysis included Kyoto Encyclopedia of Genes and Genomes (KEGG) pathways. The top 12 enriched categories were determined based on a significance threshold of p < 0.05. Next, differentially expressed proteins were input into the STRING website (https://string-db.org/) to obtain a protein–protein interaction (PPI) network with a cut-off of p < 0.05 and an interaction score > 0.4. Subsequently, the obtained PPI network was imported into Cytoscape v3.7.1 software, and hub genes were calculated using the cytoHubba plugin with MCC, MNC, EPC, Degree, and Closeness algorithms. The intersection of hub genes was determined using the Venn diagram method. To further explore the phenotypic effect and mechanism of MFN2 in HR, we used gene set enrichment analysis (GSEA v.4.3.0, Broad Institute, Massachusetts Institute of Technology, and Regents of the University of California, USA) software and DAVID website (https://david.ncifcrf.gov/) to perform pathway enrichment analysis for OE-MFN2 in proteomics sequencing data. For the GSEA analysis, the default parameters of the software were selected. Then, the relevant pathways and phenotypic proteins in the enrichment analysis results were selected for correlation analysis and display through PerformanceAnalytics R package.

### Statistical data analysis

The data were statistically analyzed using GraphPad Prism v.9.0. Data are shown as mean ± SD. Statistical analysis was conducted using two-tailed Student's t-tests to compare two groups. A one-way analysis of variance (ANOVA) was used for multi-group comparison. Results with a p-value less than 0.05 were regarded as statistically significant.

## Result

### Isolation and verification of MAMs from HK-2 cells

To elucidate the MAM profile in acute kidney injury, we firstly isolated MAMs from human HK-2 cells for proteomics analysis, according to a well-established method as shown in Additional file 1: Fig. S1.

A Western blot was used to confirm the purity of MAMs isolated by using several organelle marker proteins (Additional file 2: Fig. S2). Mitofusin2(MFN2) [31] and Glucose-regulated protein 75 (GRP75) [32] was reported to be enriched in MAMs fraction, both of which presented at comparable levels in our MAMs and pure mitochondria(PM) fractions and MFN2 was also detected in the ER. β-actin as a cytosolic marker should be absent in MAMs, PM and ER. VDAC1 and TOM20, located in the outer mitochondrial membrane, were found to be relatively low expressed in MAMs in our results. Conversely, COXIV and Cytochrome C (CytC), situated in the inner mitochondrial membrane, demonstrated distinct distribution patterns. COXIV exhibited a minor presence primarily in the PM and in limited quantities in MAMs. On the other hand, CytC was exclusively observed in the pure mitochondria (PM). IP3R, is commonly acknowledged MAM and ER markers [28]. Thus, these data suggest that the MAMs we isolated was purely and reproducibly.

### Characterization of total MAM proteins in HK-2 cells

**Fig. 1:**
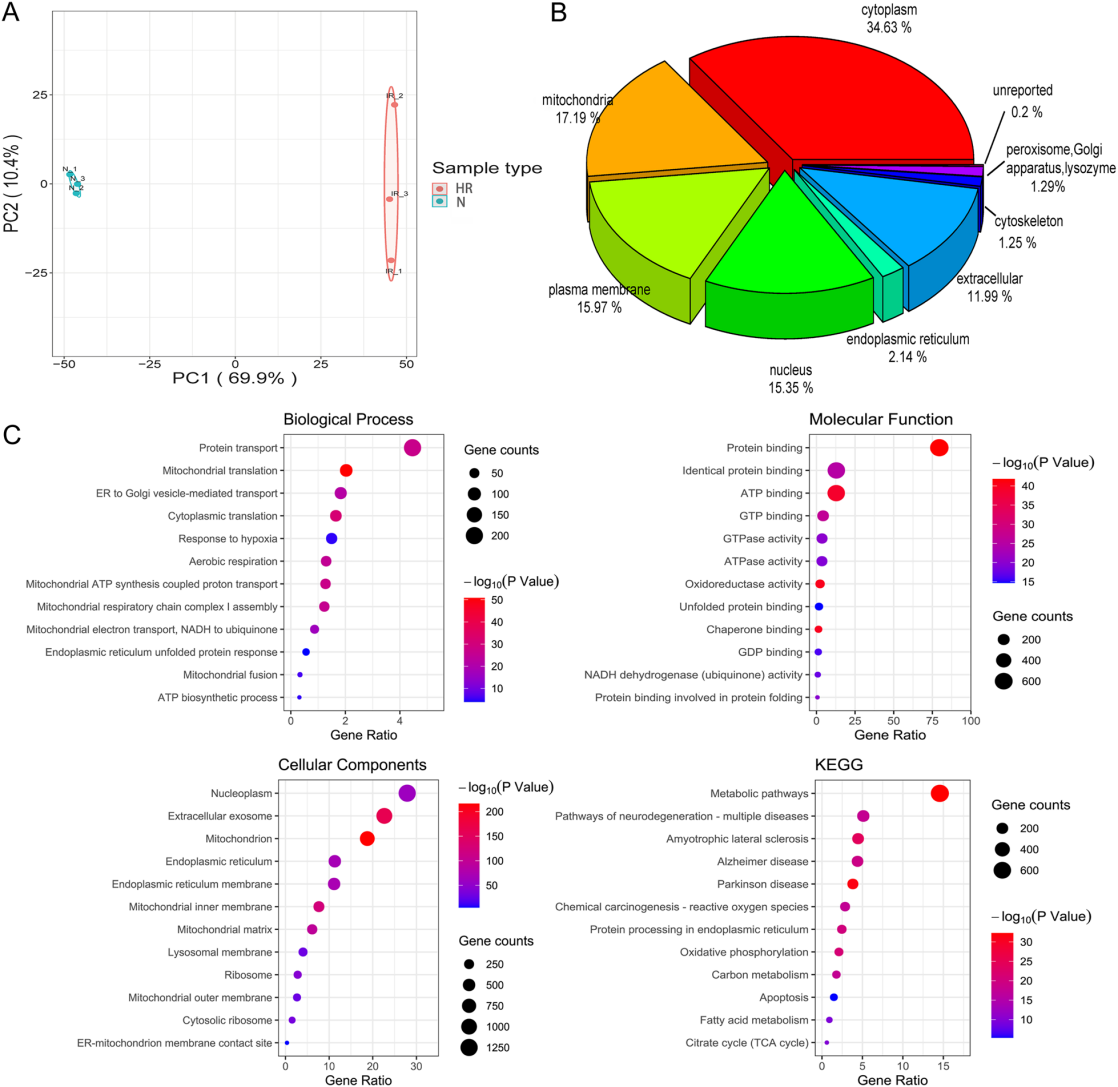
Comprehensive characterization of total MAM proteins in HK-2 cells. **A** Principal component analysis (PCA) of the proteome between two groups. The principal component analysis (PCA) plot demonstrates a high level of reproducibility within the three samples in one group, as well as a significant degree of distinction between normal groups (N) and hypoxia/reoxygenation (HR) groups, showing distinct clustering. **B** Subcellular distribution of proteins identified in total MAM fractions. **C** Gene Ontology (GO) and Kyoto Encyclopedia of Genes and Genomes (KEGG) enrichment analysis of total proteins, highlighting biological processes (BP), cellular components (CC), molecular functions (MF), and KEGG pathways; top 12 results displayed for each category

Using the DAVID website, we conducted a GO/KEGG enrichment analysis of HK-2 cell MAM proteins. The organelle localization, top 12 enrichment results for biological process (BP), cell component (CC), molecular function (MF) and KEGG pathways are plotted (Fig. 1).

The principal component analysis (PCA) plot demonstrates a high level of reproducibility within the three samples in one group, as well as a significant degree of distinction between two different groups (Fig. 1A). The total number of proteins identified was 4489, with 3531 successfully quantified. These 3531 proteins were studied using clustering analysis followed by GO and KEGG pathways. As shown in Fig. 1B, 34.63% (1557) of proteins exist in cytoplasm and 17.19% (773) proteins belonged to mitochondria; 15.97% (718) are in plasma membrane;15.35% (690) are in nucleus; 2.14% (96) are in endoplasmic reticulum (Additional file 3: Table S1). The enrichment of nucleus and plasma membrane proteins within the MAMs can be due to the dynamic interactions among the mitochondria, plasma membrane, and endoplasmic reticulum (ER) [33]. This phenomenon suggests that there are intricate connections between the ER and neighboring organelles such as the nucleus, as well as intercompartmental translocation of proteins involved in pathophysiological processes like stress, inflammation, apoptosis [34] and so on. We speculate that the presence of plasma membrane proteins in the MAMs is the active translocation and trafficking of novel proteins to and from this region because MAMs has membrane structural properties itself. These processes may play a role in signaling transduction and biogenesis.

Functionally, these proteins are implicated in biological processes and molecular function that appear to be closely related to mitochondrial function and energy metabolism (Fig. 1C, Additional file 4: Table S2). In terms of biological processes, mitochondrial translation, though not exhibiting the highest abundance of enriched proteins, does exhibit the foremost level of statistical significance in biological process(P = 1.61E−51). Other processes include, but are not limited to, mitochondrial ATP synthesis coupled proton transport, protein transport, mitochondrial respiratory chain complex I assembly, and aerobic respiration, among others. Mitochondrial fusion and endoplasmic reticulum unfolded protein response are well-established biological processes that have been extensively studied and confirmed to have close associations with MAMs. Response to hypoxia may be a biological process unique to our model that differs from previous studies. Molecular functional analysis indicates that molecules enriched in the top 12 positions are also closely associated with energy metabolism, storage, and related processes, such as ATP binding, GTP binding, GTPase activity, ATPase activity, etc. Thereinto, many kinds of molecular chaperones and oxidoreductases supposed to be localized in MAMs, which is as expected.

In the intricate network of cellular interactions, numerous crucial molecules emerge, exhibiting enrichment in specific molecular functions while simultaneously participating in one or more biological processes. For instance, NDUFA13, an accessory subunit of the mitochondrial membrane respiratory chain NADH dehydrogenase (Complex I), plays a pivotal role in sevel biological processes such as mitochondrial ATP synthesis coupled proton transport, mitochondrial respiratory chain complex I assembly, and aerobic respiration. ERO1α, a significant oxidoreductase, contributes to endoplasmic reticulum unfolded protein response. SCARB2 acts as a molecular companion involved in cholesterol metabolism and endoplasmic reticulum-mitochondria interactions. EIF2AK3, EIF2AK2, VAPB, and CANX, enriched in the endoplasmic reticulum unfolded protein response, are considered to have close associations with MAMs. This underscores their intricate roles in maintaining cellular homeostasis and responding to stress, further emphasizing their significance in cellular processes. EIF2AK3 (PERK) and EIF2AK2 (PKR) are members of the eIF2α kinase family, pivotal regulators in cellular stress responses. VAPB: a key member of the VAP protein family, acts as a tether between the ER and mitochondria, fostering membrane contact sites. CANX: A Guardian of ER Integrity Calnexin (CANX), a chaperone residing in the ER, ensures proper folding of newly synthesized glycoproteins. APP(Amyloid Precursor Protein),enriched in various molecular functions such as protein binding, chaperone binding, and identical protein binding, as observed in our study, which highlight the multifaceted role of APP in mediating protein interactions and chaperone activities within MAMs.

Mitochondrial fusion, a critical aspect of cellular homeostasis, involves a network of proteins. In our research, several key proteins are enriched, including FIS1 and MFF, primarily located in the outer mitochondrial membrane, participating in fission events. Meanwhile, SPG7 and AFG3L2 reside in the inner mitochondrial membrane, contributing to various mitochondrial functions [35]. OPA1, MFN1, and MFN2 participate in fusion processes, influencing the integrity of both inner and outer mitochondrial membranes. Additionally, BAX and BAK1, located in the outer mitochondrial membrane, play essential roles in the regulation of apoptosis. This intricate protein network, with specific mitochondrial localizations and functions, collectively orchestrates mitochondrial dynamics, contributing to overall cellular health.

To identify the top 12 biological pathways statistically significant, KEGG analysis was conducted. Among them, the metabolic pathways ran the first, indicating the role of mitochondria metabolism to the MAM function. Many other KEGG pathways are related to the nervous system disease, such as pathways of neurodegeneration, amyotrophic lateral sclerosis, Alzheimer disease, Parkinson disease. Additionally, several other significant pathways are among them, including chemical carcinogenesis-reactive oxygen species, protein processing in the endoplasmic reticulum, oxidative phosphorylation, carbon metabolism, the citrate cycle, and so on. These findings broaden our understanding of the molecular function of MAMs in kidney.

### Characterization of altered MAM proteins in HR HK-2 cells

**Fig. 2:**
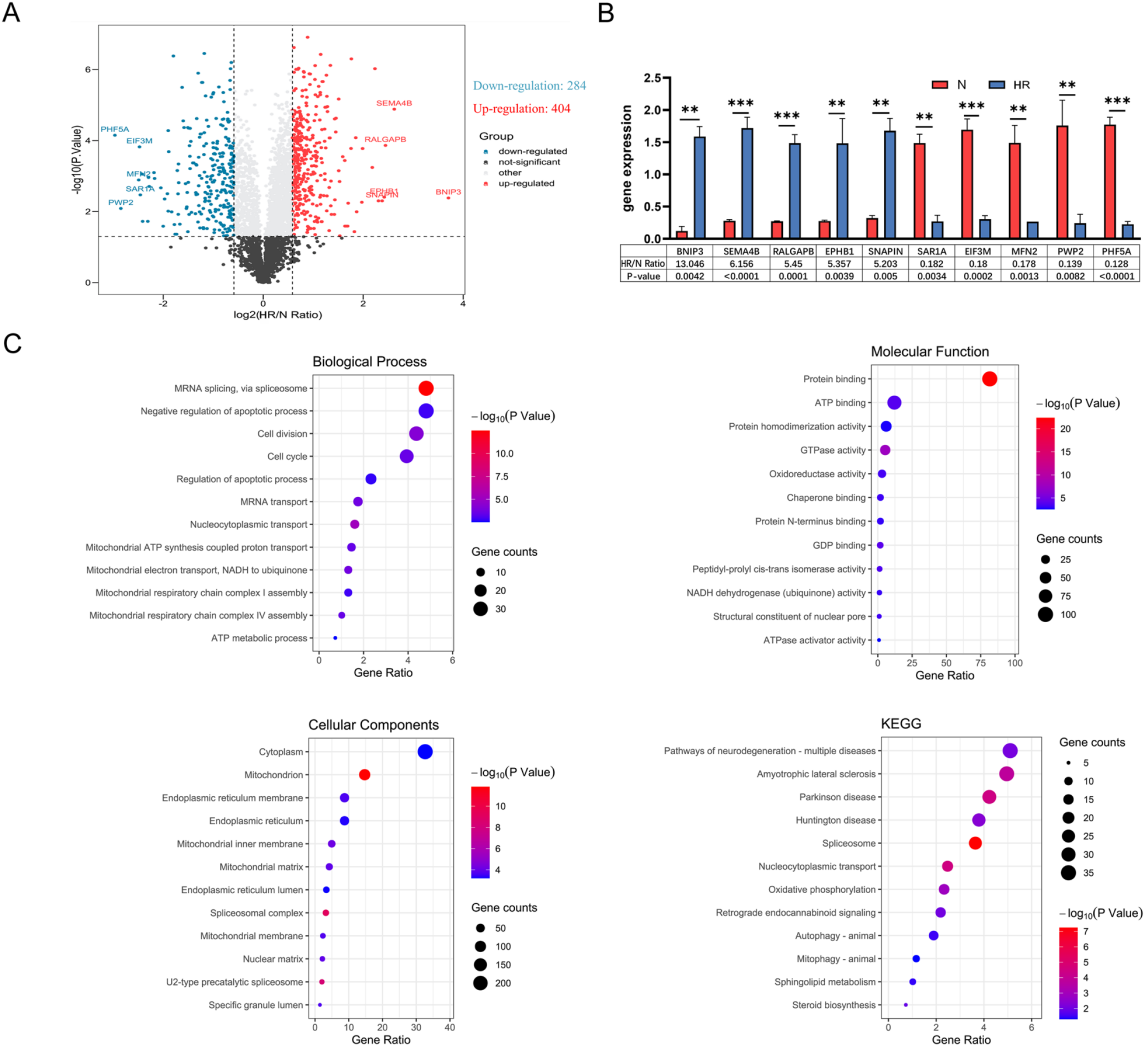
Differentially expressed proteins and enrichment analysis in N and HR groups. **A** Volcano plot depicting differential protein expression between N and HR groups, with −log10 (p-value) versus log2 (HR/N ratio). **B** Top 10 proteins with significant abundance changes in HR/N comparison.BNIP3 is associated with potential engagement in mitochondrial autophagy; SEMA4B may contribute to altered cell migration and apoptosis; RALGAPB implies regulatory involvement in cellular processes through GTP hydrolysis; EPHB1, as a tyrosine kinase, may signify engagement in signaling pathways crucial for cellular adaptation; SAR1A, EIF3M, and PWP2 may affect protein transport and synthesis, respectively; PHF5A may affect mRNA splicing, MFN2:mitochondrial outer membrane protein that participates in mitochondrial fusion and contributes to the maintenance and operation of the mitochondrial network. **C** GO/KEGG enrichment analysis of differentially expressed proteins; top 12 results for BP, CC, MF, and KEGG pathways displayed. Data expressed as mean ± SEM; significance denoted as *p < 0.05, **p < 0.01, ***p < 0.001

We conducted an in-depth analysis of the MAM proteome to gain a better understanding of effect during HR. In our analysis of altered MAM proteins in HR HK-2 cells, we observed a notable number of proteins exhibiting significant changes in their expression levels. This suggests a dynamic response within the MAMs under HR conditions. Functionally, these alterations likely impact key cellular processes associated with mitochondrial function, energy metabolism, and interorganellar communication. Among the total quantifiable proteins, 688 proteins in HR HK-2 cells exhibited significant changes in expression level with P-value < 0.05 and HR/N > 1.5 or HR/N < 0.66 set as the threshold criteria. Of these, the abundance of 404 proteins significantly increased, while the abundance of 282 proteins significantly decreased. To visually illustrate the significance and magnitude of protein changes, volcano plot −log10 (P value) vs.log2 (HR/N Ration)—was constructed (Fig. 2A).

The top 10 entries in the HR/N ratio represent the mean protein abundances and the variability among the individual samples (Fig. 2B). Upregulated molecules include BNIP3, SEMA4B, RALGAPB, EPHB1, and SNAPIN, while downregulated molecules comprise SAR1A, EIF3M, MFN2, PWP2, and PHF5A.BNIP3 showed the highest protein abundance change in HR/N (13.046).

The major cellular localizations of 688 proteins are cytoplasm (46.95%, 323 proteins), mitochondria (15.26%, 105 proteins), plasma membrane (14.24%, 98 proteins), nucleus (6.98%, 48 proteins), endoplasmic reticulum (2.18%, 15 proteins), and so on (Additional file 3: Table S1).

We further analyzed the 688 differentially expressed MAM proteins into their respective functional categories using above methods (Fig. 2C). The results showed that changes in biological process were significantly enriched in MRNA splicing, negative regulation of apoptotic process, cell division, cell cycle, regulation of apototic process, nucleocytoplasmic transport et al. Changes in the molecular function were mainly enriched in protein binding, ATP binding, protein homodimerization activity, GTPase activity, oxidoreductase activity, chaperone binding, et al. Changes in cellular component were mainly enriched in cytoplasm, mitochondrion, endoplasmic reticulum membrane, endoplasmic reticulum, mitochondrial inner membrane, mitochondrial matrix, et al. Our enrichment analysis indicates that the primary molecular functions of differentially expressed proteins post hypoxia/reoxygenation align with the total protein pool, predominantly associated with mitochondrial energy metabolism. This concurrence with established functions of MAMs in previous research is notable. However, distinctive differences emerge when considering biological processes and molecular functions. Differentially expressed proteins post hypoxia/reoxygenation are enriched in biological processes such as apoptotic processes, cell division, cell cycle, and regulation of apoptotic processes—processes typically associated with cellular damage. In contrast to the total protein pool, these findings suggest a specific response of certain proteins to hypoxic/reoxygenation, potentially playing crucial roles in the regulation of these cellular processes.

Furthermore, KEGG pathway revealed that the top 4 significantly enriched terms were involved in neurodegenerative disease, including pathways of neurodegeneration-multiple diseases, amyotrophic lateral sclerosis, Parkinson disease, Huntington disease. Spliceosome, consistent with mRNA splicing in biological process, shows the highest significance (p = 5.84E−08, Additional file 5: Table S3). This result is consistent with previous studies in testis proteome of Wang [36], suggesting that spliceosome may have a close relationship with MAMs. Oxidative phosphorylation, as an important part of metabolic process, is thought to play an important role in ischemic reperfusion injury [37]. Autophagy and mitophagy, are also believed to play a crucial part in renal ischemic reperfusion injury.

In summary, our study's strengths encompass a comprehensive characterization of MAMs, the identification of distinct proteins, pathways, and interactions MAMs in the context of HR represents a novel and insightful contribution to the field.

### Protein–protein interaction network and hub genes

**Fig. 3:**
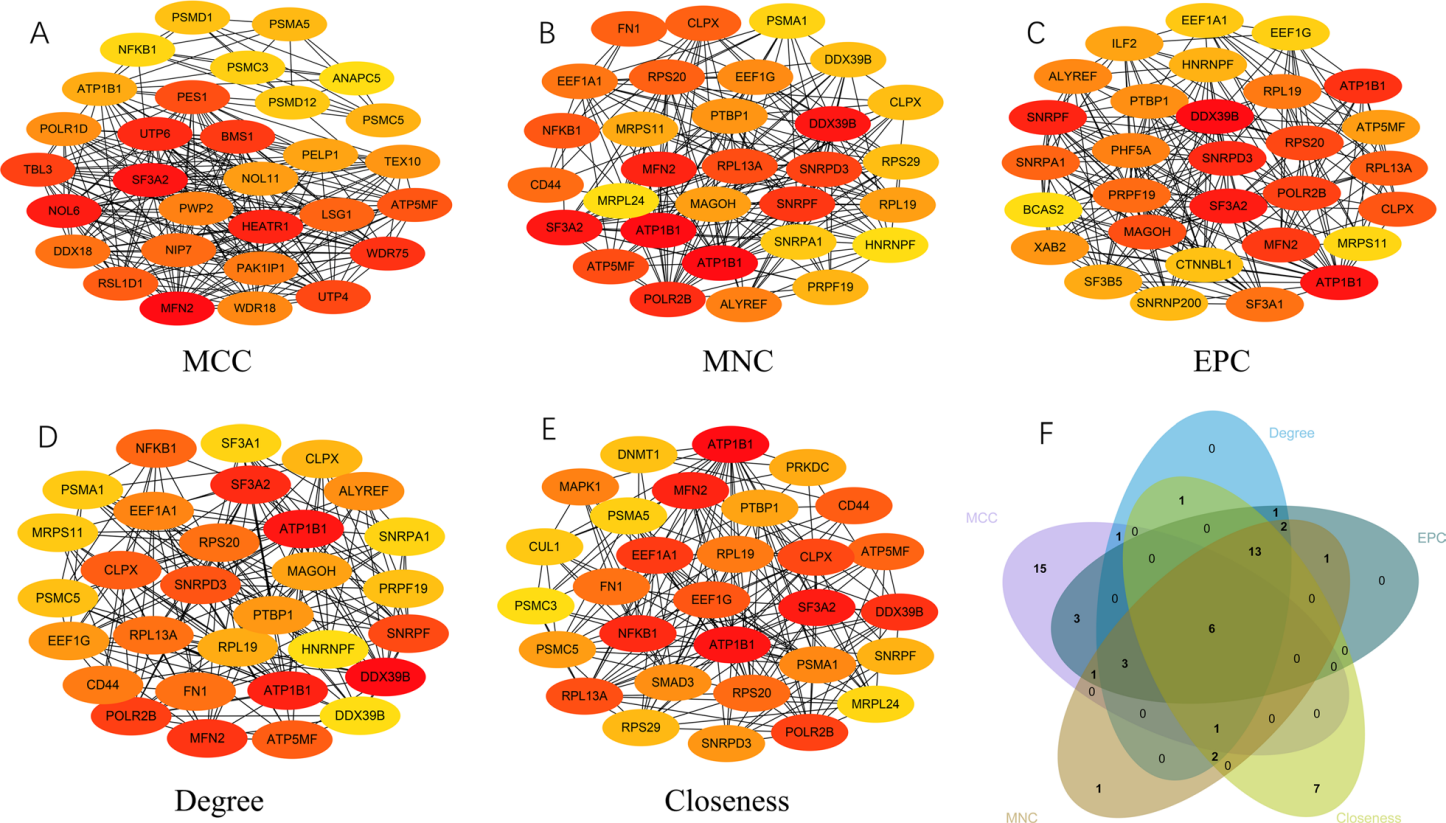
Establishment of the protein–protein interaction (PPI) network. **A**–**E** Identification of top 30 central genes in PPI network via MCC, MNC, EPC, Degree, and Closeness algorithms. **F** Venn diagram showing common gene identified across all five algorithms

In order to gain a deeper comprehension of these distinctively expressed proteins, we utilized the "STRING" application within the Cytoscape software to construct a protein–protein interaction (PPI) network (Fig. 3A–F). The network was established by selecting the top 30 items from each algorithm using the cytoHubba plug-in （Additional file 6：Table S4). To enhance credibility and reliability, we calculated the intersection of genes obtained from five algorithms, as previous studies [38]. The resulting genes include MFN2, SF3A2, DDX39B, ATP1B1, CLPX, and ATP5MF. Among them, ATP1B1, CLPX, and ATP5MF have been reported to be associated with cellular protein metabolic processes. MFN2 is a multifunctional protein primarily involved in mitochondrial fusion, which was ranked in the top 10 differential proteins. SF3A2 and DDX39B predominantly engage in the splicing process. All of these identified hub genes exhibit a significant enrichment in crucial biological functions and KEGG pathways, closely associated with differentially expressed genes in response to HR.

### Changes of actin cytoskeleton in MAMs

Mito-ER contact sites are intricately regulated by the actin cytoskeleton, driving fission and mitochondrial dynamics. The actin cytoskeleton serves as a pivotal force in governing ER-mitochondrial function and interaction, exerting its influence across various cellular aspects, including maintaining cell morphology, regulating organelle distances, modulating signal transduction, and preserving cellular functionality. In our quantitative proteomic analysis, a total of 25 actin or actin-related proteins were identified in the quantifiable protein pool (Table 1). Among these, four were associated with differentially expressed genes: ACTR8 was upregulated, while the other three genes (ARPC2, CAPZA2, INF2) were downregulated. These proteins may play a role in regulating the structure and function between mitochondria and the endoplasmic reticulum.

**Table 1:** Actin and actin-related proteins in MAMs

Protein accession	Protein description	Gene name	HR/N Ratio	HR/N P value
P52907	F-actin-capping protein subunit alpha-1 OS = Homo sapiens OX = 9606 GN = CAPZA1 PE = 1 SV = 3	CAPZA1	0.675	1.91354E−05
Q9H981	Actin-related protein 8 OS = Homo sapiens OX = 9606 GN = ACTR8 PE = 1 SV = 2	ACTR8	1.743	2.44979E−05
O15144	Actin-related protein 2/3 complex subunit 2 OS = Homo sapiens OX = 9606 GN = ARPC2 PE = 1 SV = 1	ARPC2	0.629	0.000162005
P47755	F-actin-capping protein subunit alpha-2 OS = Homo sapiens OX = 9606 GN = CAPZA2 PE = 1 SV = 3	CAPZA2	0.514	0.000240551
Q9UPN3	Microtubule-actin cross-linking factor 1, isoforms 1/2/3/5 OS = Homo sapiens OX = 9606 GN = MACF1 PE = 1 SV = 4	MACF1	0.831	0.000266357
Q9H2D6	TRIO and F-actin-binding protein OS = Homo sapiens OX = 9606 GN = TRIOBP PE = 1 SV = 3	TRIOBP	1.34	0.000888008
O94851	[F-actin]-monooxygenase MICAL2 OS = Homo sapiens OX = 9606 GN = MICAL2 PE = 1 SV = 1	MICAL2	0.72	0.000983326
P12814	Alpha-actinin-1 OS = Homo sapiens OX = 9606 GN = ACTN1 PE = 1 SV = 2	ACTN1	1.194	0.001054492
P47756	F-actin-capping protein subunit beta OS = Homo sapiens OX = 9606 GN = CAPZB PE = 1 SV = 4	CAPZB	0.873	0.00123045
Q13561	Dynactin subunit 2 OS = Homo sapiens OX = 9606 GN = DCTN2 PE = 1 SV = 4	DCTN2	1.408	0.001352673
Q9BPX5	Actin-related protein 2/3 complex subunit 5-like protein OS = Homo sapiens OX = 9606 GN = ARPC5L PE = 1 SV = 1	ARPC5L	0.82	0.001545111
Q9BPX5	Actin-related protein 2/3 complex subunit 5-like protein OS = Homo sapiens OX = 9606 GN = ARPC5L PE = 1 SV = 1	ARPC5L	0.82	0.001545111
Q14203	Dynactin subunit 1 OS = Homo sapiens OX = 9606 GN = DCTN1 PE = 1 SV = 3	DCTN1	0.891	0.002386974
P59998	Actin-related protein 2/3 complex subunit 4 OS = Homo sapiens OX = 9606 GN = ARPC4 PE = 1 SV = 3	ARPC4	0.827	0.003001207
P23528	Cofilin-1 OS = Homo sapiens OX = 9606 GN = CFL1 PE = 1 SV = 3	CFL1	1.241	0.005974978
Q27J81	Inverted formin-2 OS = Homo sapiens OX = 9606 GN = INF2 PE = 1 SV = 2	INF2	0.619	0.030143459
P61160	Actin-related protein 2 OS = Homo sapiens OX = 9606 GN = ACTR2 PE = 1 SV = 1	ACTR2	0.924	0.035312039
Q9GZN1	Actin-related protein 6 OS = Homo sapiens OX = 9606 GN = ACTR6 PE = 1 SV = 1	ACTR6	1.24	0.047491029
Q9GZN1	Actin-related protein 6 OS = Homo sapiens OX = 9606 GN = ACTR6 PE = 1 SV = 1	ACTR6	1.24	0.047491029
P61158	Actin-related protein 3 OS = Homo sapiens OX = 9606 GN = ACTR3 PE = 1 SV = 3	ACTR3	0.949	0.112223733
O15145	Actin-related protein 2/3 complex subunit 3 OS = Homo sapiens OX = 9606 GN = ARPC3 PE = 1 SV = 3	ARPC3	0.95	0.133295535
P68133	Actin, alpha skeletal muscle OS = Homo sapiens OX = 9606 GN = ACTA1 PE = 1 SV = 1	ACTA1	0.872	0.137965167
Q92747	Actin-related protein 2/3 complex subunit 1A OS = Homo sapiens OX = 9606 GN = ARPC1A PE = 1 SV = 2	ARPC1A	0.88	0.210845143
O15511	Actin-related protein 2/3 complex subunit 5 OS = Homo sapiens OX = 9606 GN = ARPC5 PE = 1 SV = 3	ARPC5	0.903	0.597673208
O15143	Actin-related protein 2/3 complex subunit 1B OS = Homo sapiens OX = 9606 GN = ARPC1B PE = 1 SV = 3	ARPC1B	0.988	0.618529405

### Validation of BNIP3 and MFN2 Changes with qRT-PCR and WB

**Fig. 4:**
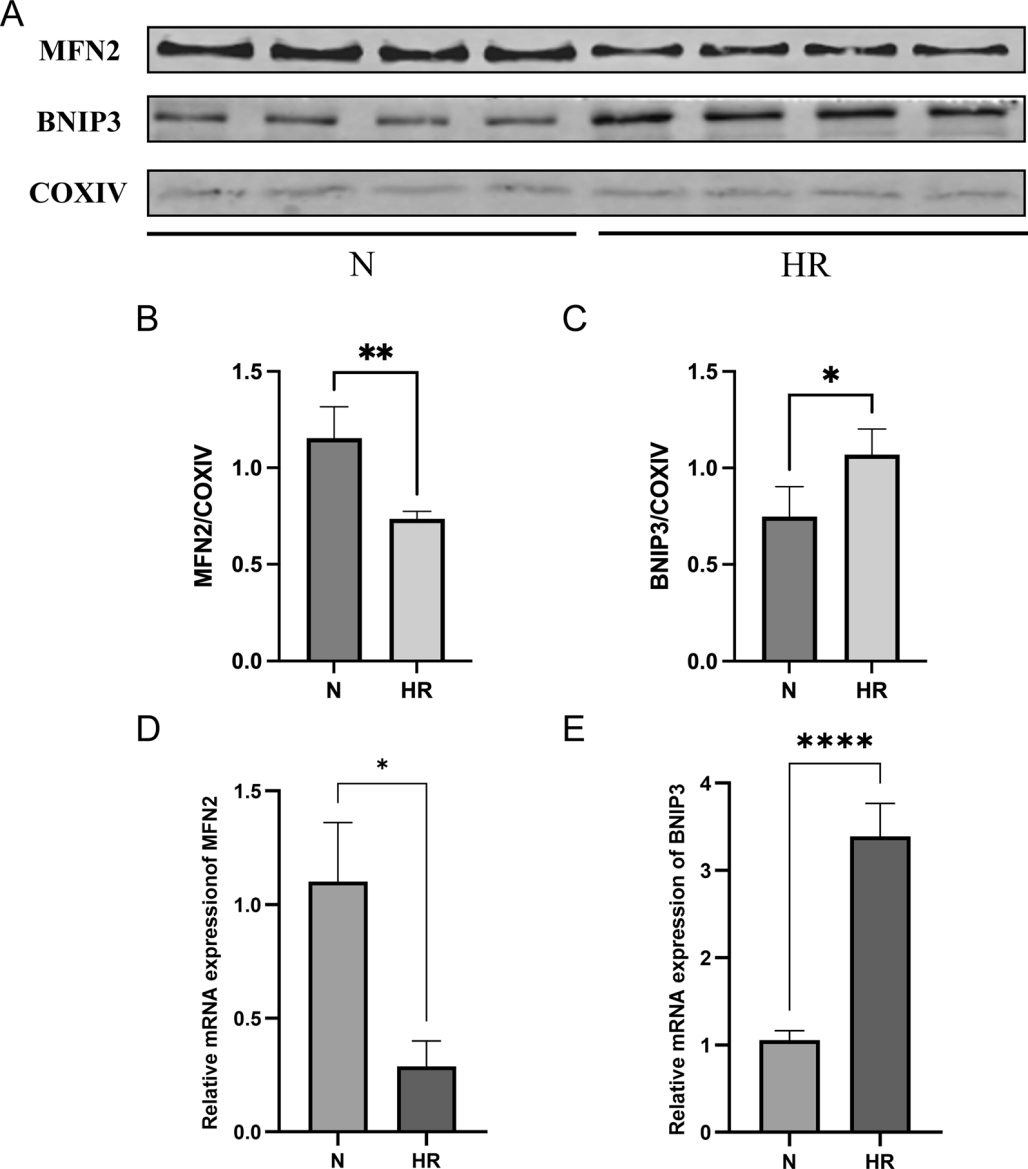
Validation of BNIP3 and MFN2 alterations via western blot (WB) and quantitative real time polymerase chain reaction (qRT-PCR). **A** Densitometric analysis of MFN2, BNIP3, and CoxIV bands. **B**, **C** Relative quantification of MFN2: COXIV and BNIP3: COXIV ratios, indicating significant changes in HR group versus N group. **D**, **E** mRNA expression levels of MFN2 and BNIP3. Data expressed as mean ± SEM; significance denoted as *p < 0.05, **p < 0.01, ***p < 0.001, ****p < 0.0001

Among the mass spectrometry identified MAM proteins in the top 10 entries of HR/N ratio, we selected two of them changes for qRT-PCR and WB validation (Fig. 4). One of them was BNIP3, which showed the highest degree of change (13.046 in HR/N). The other was MFN2, which was one of the hub genes and was considered to be one of most important proteins closely related to MAMs and mitochondrial dynamics. As indicated in Fig. 4D, E, exposure to HR markedly decreased the mRNA expression of MFN2 compared to the normal group under normoxic conditions. In contrast, the mRNA levels of BNIP3 were upregulated. Western blot validation corroborated these findings, demonstrating consistency between mRNA and protein expression. MAMs markers such as GRP75, VDAC1 may change under hypoxic conditions. We chose COXIV as the reference, although it is very lowly abundant in MAMs. Using ImageJ software, the densitometry data of the BNIP3 and MFN2 protein bands were normalized to those of COXIV. Results showed that BNIP3 was significantly upregulated, while MFN2 was significantly downregulated, which was consistent with our proteomic results.

### Validation of in vivo experiments

**Fig. 5:**
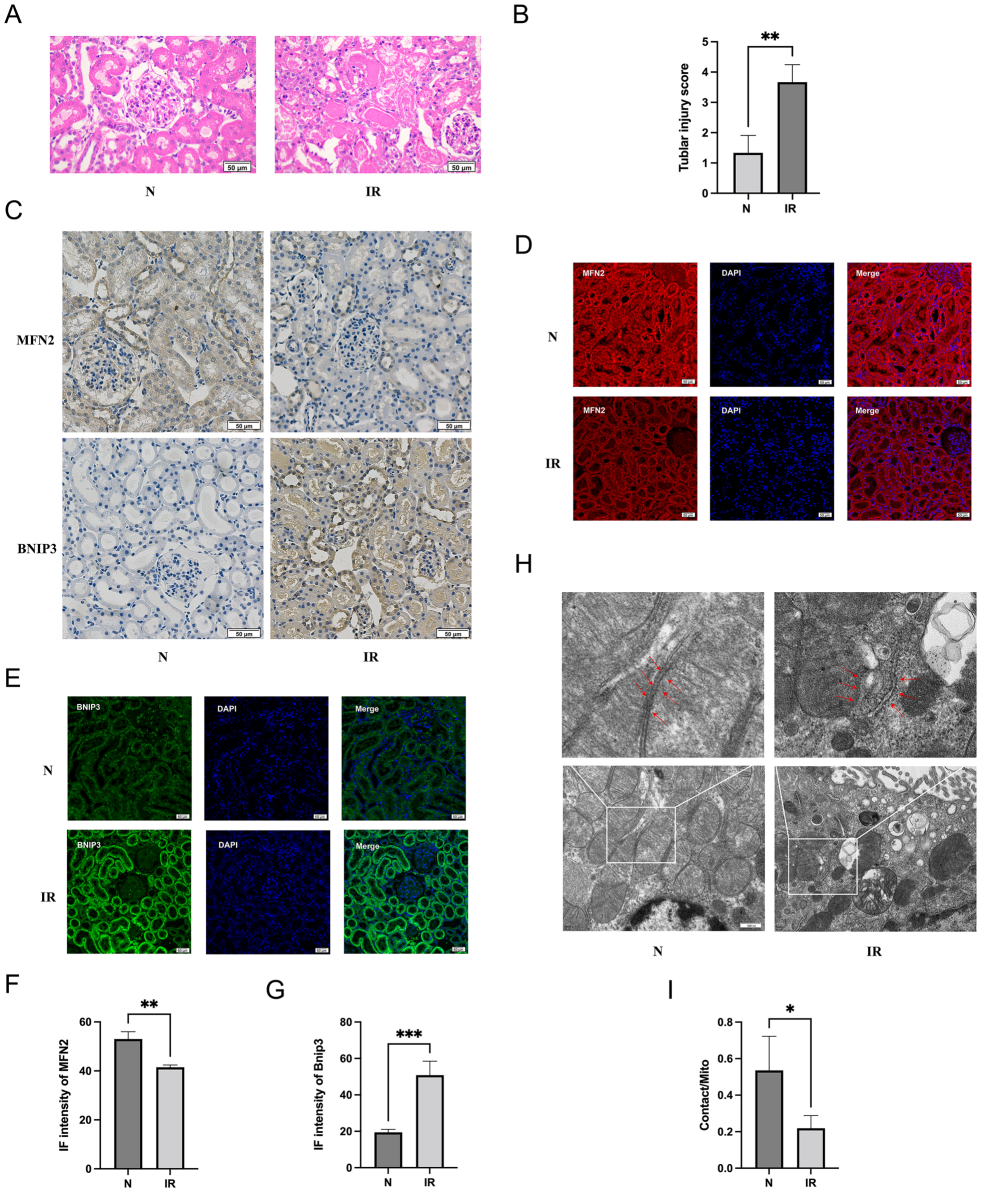
Validation experiments in vivo. **A**, **B** HE staining and renal pathology scoring in normal and ischemic reperfusion kidneys. **C** Immunohistochemical staining for MFN2 and BNIP3 in rat kidney tissues. **D**–**G** Immunofluorescence staining for MFN2 and BNIP3. **H**, **I** Transmission electron microscopy of rat kidney MAMs. Data expressed as mean ± SEM; significance denoted as *p < 0.05, **p < 0.01, ***p < 0.001

In the in vivo rat experiments of IRI, histopathological assessments using hematoxylin and eosin (H&E) staining indicated significant tubular damage. (Fig. 5A, B) Immunohistochemistry (IHC) and immunofluorescence (IF) analyses further revealed a downregulation of MFN2 and an upregulation of BNIP3 expression in renal tubules, consistent with the proteomic findings in vitro. (Fig. 5C–G).

Moreover, electron TEM results of MAMs mirrored the outcomes observed in cellular experiments. Following renal IRI, an increase in mitochondrial fission events was evident, accompanied by a reduction in MAMs. These morphological changes align with the alterations identified in the proteomic analysis, collectively highlighting the impact of ischemic reperfusion injury on mitochondrial dynamics and MAMs composition in renal tissues (Fig. 5H, I).

### The reduction of MAMs in HR HK-2 cells was reversed by MFN2 overexpression, whereas MFN2 knockdown exacerbated the damage

**Fig. 6:**
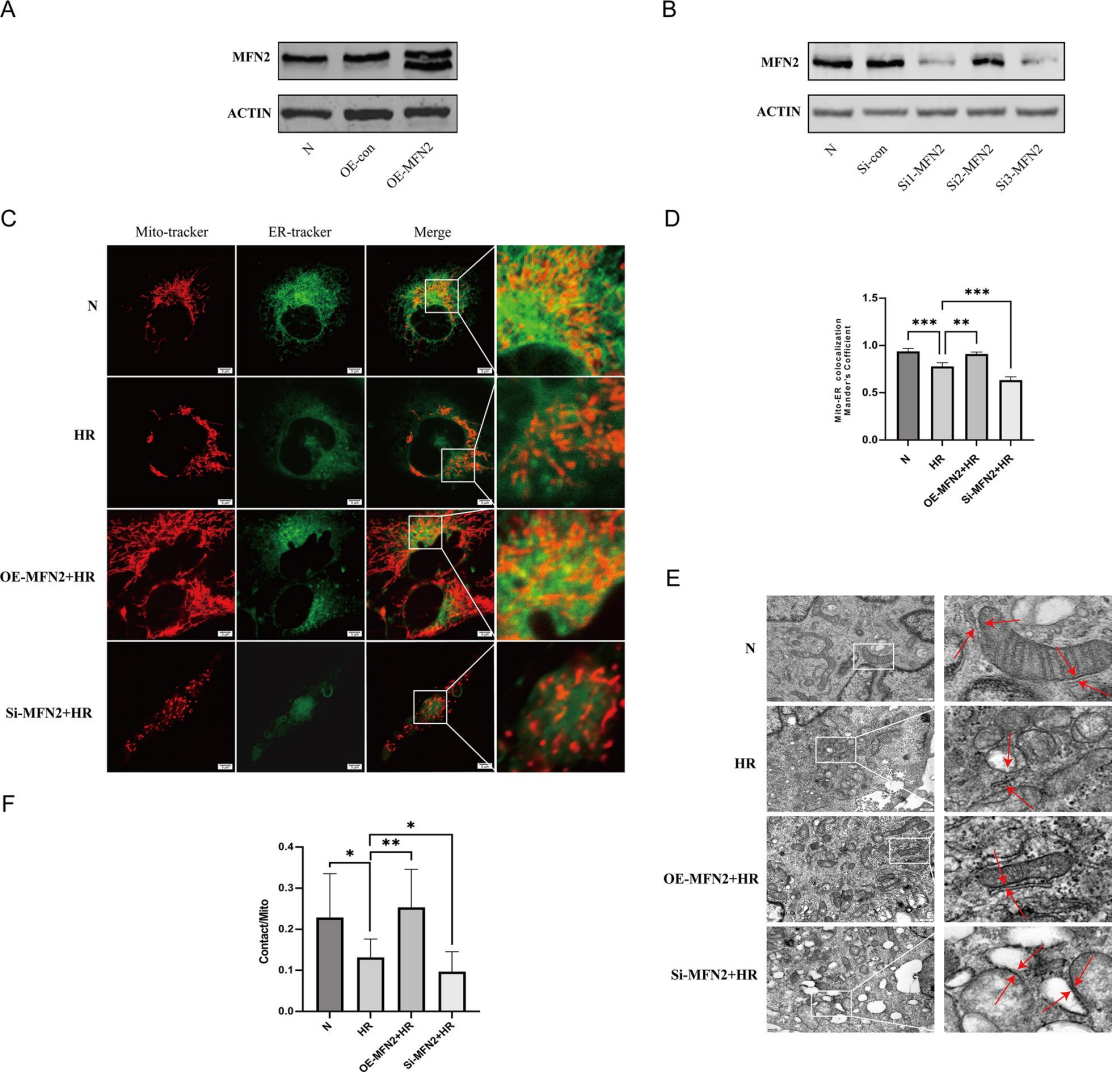
MAM dynamics in HK-2 cells under various conditions. **A** Increased MFN2 expression induced by OE-MFN2. **B** Reduced MFN2 expression via Si-MFN2. **C**, **D** Staining of HK-2 cells under N, HR, HR + OE-MFN2 and HR + Si-MFN2 conditions using MitoTracker and ER Tracker, quantification of Manders' coefficient for mitochondria-ER overlap. **E**, **F** Transmission electron microscopy analysis of MAMs and mitochondrial damage. Significance denoted as *p < 0.05, **p < 0.01, ***p < 0.001

To assess the impact of MFN2 on MAMs in ischemic reperfusion (IR), we conducted in vitro experiments using HK-2 cells subjected to hypoxia/reoxygenation treatment with modulation of MFN2 expression. The Manders' colocalization coefficient in the HR group exhibited a significant decrease, indicating reduced mitochondria-ER contact. Overexpression of MFN2 (OE-MFN2) reversed this reduction, while knockdown of MFN2(Si-MFN2) further decreased the Manders' colocalization coefficient. Moreover, HR exacerbated mitochondrial fission, which was counteracted by an increase in mitochondrial fusion in the OE-MFN2 group, while Si-MFN2 exacerbated mitochondrial fission. Transmission electron microscopy confirmed these observations, showing increased mitochondrial fragmentation and instances of swelling or edema in the HR group. Additionally, HR-induced a significant decrease in MAMs compared to the Normal group, a phenomenon reversed by MFN2 overexpression but further aggravated by Si-MFN2 (Fig. 6).

### Proteomics analysis of OE-MFN2 and KEGG pathway exploration and verification

**Fig. 7:**
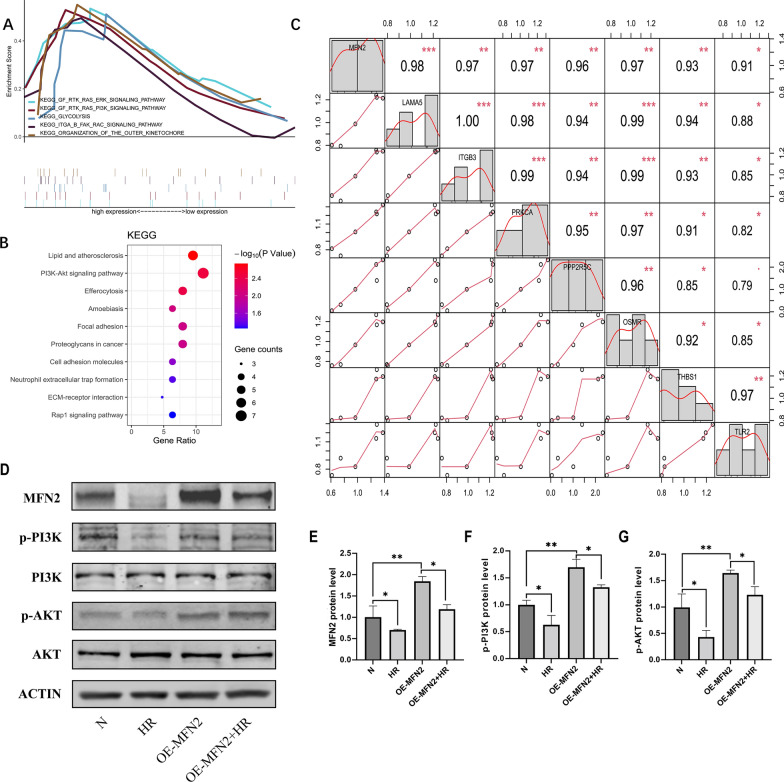
MFN2 regulation of MAM-related mechanisms in HK-2 cells. **A** Gene set enrichment analysis (GSEA) of proteomics in normal versus OE-MFN2 HK-2 cells. **B** KEGG analysis of upregulated genes in OE-MFN2 HK-2 cell proteomics. **C** Correlation analysis of key molecules in the PI3K/AKT pathway with MFN2. **D**–**G** Expression levels of MFN2, PI3K, p-PI3K, AKT, and p-AKT. Data expressed as mean ± SEM; significance denoted as *p < 0.05, **p < 0.01, ***p < 0.001

To further elucidate the role of MFN2 in renal tubules and understand its mechanisms and associated pathways, we conducted a proteomic study on normal HK-2 cells and MFN2 overexpressing (OE-MFN2) HK-2 cells. GSEA analysis was performed using GSEA 4.3.2 software (https://www.gsea-msigdb.org/gsea/index.jsp). Additionally, we subjected the upregulated genes in the OE-MFN2 group to DAVID enrichment analysis, followed by visualization using R software. KEGG enrichment results revealed a significant positive correlation between the PI3K pathway and MFN2. Upon literature review and considering the aforementioned analyses, we conducted correlation analysis between upregulated genes in the PI3K pathway and MFN2. The results identified genes within the PI3K/AKT pathway, including LAMA5, ITGB3, PRKCA, PPP2R5C, OSMR, THBS1, and TLR2, showing a correlation with MFN2 (Fig. 7A–C).

Subsequently, we validated the relationship between OE-MFN2 and N group in the PI3K/AKT pathway during HK-2 cell HR. The results demonstrated a positive regulatory association between the PI3K/AKT pathway and MFN2, consistent with the findings from the proteomic KEGG analysis. The results of our study provide extensive information about MAM protein changes after renal ischemic reperfusion injury, which is helpful for understanding mechanisms of this condition (Fig. 7D–G).

## Discussion

### Overview of MAMs proteomics

Proteomics is one of the important approaches to exploring membrane proteins comprehensively. By employing advanced 4D label-free mass spectrometry (MS) analysis, particularly enabling the identification of a large number of non-redundant proteins, we investigated changes in the proteome profile of MAMs isolated from HK-2 cells. MAM proteins differentially expressed were identified through bioinformatics analysis in normal and hypoxia/reoxygenation model of HK-2 cells. Our findings may underscore the vital role of MAMs in renal ischemic reperfusion injury (IRI), particularly in association with mitochondrial energy metabolism and key biological processes such as apoptosis, division, and the cell cycle. Notably, the crucial MAM protein MFN2 may play a pivotal protective role in renal IRI.

An initial proteomic study of MAM was published in 2011, identifying 991 unique proteins by using SILAC quantitation [39]. After that, a few of studies have been performed on MAM proteomics, such as the cerebral cortex of mouse in Alzheimer’s Disease model [40], retina and brain MAMs isolated from diabetes mellitus mice [41, 42],and so on. Currently, research on MAM proteomics has primarily concentrated on neurological disorders, with relatively limited exploration in other fields, particularly in the context of organ ischemic reperfusion injury. Our study focused on MAMs in the context of renal ischemic reperfusion injury, shedding light on alterations in protein composition and potential functional implications.

Some scholars have posited that the function of MAMs plays a crucial role in organ ischemic reperfusion injury, encompassing oxidative stress, endoplasmic reticulum (ER) stress, mitophagy, calcium overload, among other factors. Nevertheless, a notable research gap exists, as no studies have systematically analyzed the proteomic changes of MAMs in renal ischemic reperfusion injury. Utilizing mass spectrometry-based proteomic analyses, our study identified MAM proteins in human HK-2 cells, investigating changes following hypoxia/reoxygenation injury, a model simulating renal ischemic reperfusion stress. The data obtained in our study not only contribute to filling this research void but also lay the foundation for a hypothesis regarding the initial phase of kidney ischemic reperfusion injury.

### Molecular function of MAM Proteins in normal and HR HK-2 cells

In the present study, we have identified 4489 non-redundant proteins in HK-2 cells MAMs with 3531 quantifiable proteins in both HR and normal MAMs, among which 688 proteins significantly altered in HR HK-2 ells with ultra-high quantitative confidence. Several proteins that have been well-studied to localize in the MAMs were detected, including ACSL4, ACAT1, ERP44, ERO1ɑ, erlin1, VAPB, CANX, MFN2 and GRP75 and VDACs. Nonetheless, PACS2, as a MAM marker, was not detected in our study, which is consistent with Zhang [39]. This observation could potentially be attributed to their comparatively limited prevalence within the MAM proteome, their suboptimal retention efficiency during MAM fractionation, or their potential interference with the detection of other proteins. Simultaneously, the expression of PACS2 within the renal tubules is relatively low [26].

Consistent with previously reported molecular functions of MAM fraction, our functional annotation of the MAM proteins suggest that MAMs is heavily involved in energetic metabolism process, such as ATP binding, GTP binding, GTPase activity, ATPase activity. In our study, both total protein and differentially expressed proteins consistently point to the close association of MAMs with mitochondrial energy metabolism. This manifest the strong association between mitochondrial energy metabolism and renal ischemic reperfusion injury, a connection that has been validated in numerous studies [43–45].Considering the kidney's role as one of the organs with the highest oxygen consumption and the fact that IRI results from damage caused by energy deprivation and subsequent reperfusion, our findings suggest that MAMs play a crucial role in both the baseline energy requirements of the kidney in physiological states and under the pathological conditions of IRI. Oxidoreductase (ERO1ɑ, ERP44 et al.) and chaperone (GRP75, CypD) are two kinds of proteins that have been substantiated by a few studies to play an important role in MAMs, ranking second and third respectively in their significance (p = 5.94E−41, 1.08E−40 respectively) in total protein, although not being the most abundant proteins enriched within these structures. In a similar vein, the functional enrichment analysis revealed that oxidoreductase activity and chaperone binding remain prominently enriched among differentially expressed proteins. Oxidoreductases, implicated in electron transfer, serve as potential indicators of alterations in cellular redox balance—a phenomenon intricately linked to both oxidative stress and mitochondrial function. Concurrently, the observed enrichment of chaperones, critical for protein folding and stability, signifies their integral role in preserving cellular homeostasis.

However, it's noteworthy that the molecular components within this molecular function differ between total proteins and those exhibiting differential expression. This suggests that these activities might operate at different levels or through distinct mechanisms in physiological and pathological conditions. These findings underscore the dynamic and context-dependent nature of cellular processes involving MAMs, shedding light on their multifaceted roles in kidney physiology and pathology.

Statistically validated KEGG analysis unveiled that the most significant pathway was metabolic pathways in both total and differential proteins. Furthermore, several other vital metabolism-related pathways were also found to be involved, such as oxidative phosphorylation, carbon metabolism, the citrate cycle. The results suggest that mitochondria, as the most energy-productive organelles in eukaryotes, play an important metabolic role in MAM function. Not surprisingly, several KEGG pathways are related to the nervous system disease, such as pathways of neurodegeneration, amyotrophic lateral sclerosis, Alzheimer disease, Parkinson disease. The possible reasons are as follows: On the one hand, the MAM proteins are highly conserved between different species and different tissues; and on the other hand, the function of MAMs has received high attention in neurological diseases, which has been a lot of evidence to support this. As compared to brain research, ischemic reperfusion injury has fewer studies involving MAM proteins, leaving a huge research gap. It is essential to note that the mitophagy pathways, exclusively enriched in the analysis of differential proteins, encompass BCL2L13, BNIP3, ATG9A, MAP1LC3B2, EIF2AK3, RRAS2, MFN2, and RPS27A. These proteins exhibit potential involvement in mitophagy processes, a pivotal cellular mechanism responsible for selectively removing damaged or dysfunctional mitochondria. Each protein may contribute to mitophagy through distinct mechanisms: BCL2L13 engages in the crosstalk between apoptosis and mitophagy; BNIP3, a key regulator, facilitates recognition and clearance of damaged mitochondria; ATG9A contributes to autophagosome formation; EIF2AK3 links mitophagy to the unfolded protein response (UPR); MFN2, beyond its role in mitochondrial dynamics, coordinates mitophagy; and RPS27A, implicated in ubiquitination, may mark mitochondria for degradation through the ubiquitin–proteasome system. This exclusive enrichment underscores their potential significance in the cellular response to hypoxic stress, emphasizing their multifaceted roles in renal ischemic reperfusion injury.

Within the 688 differentially expressed proteins between the HR and N groups, the top five upregulated molecules were BNIP3, SEMA4B, RALGAPB, EPHB1, and SNAPIN, which may signify efforts to enhance cellular resilience and combat stress-induced damage. Conversely, the top five downregulated molecules were SAR1A, EIF3M, MFN2, PWP2 and PHF5A respectively, which may point to potential vulnerabilities in cellular processes. Among them, BNIP3 is associated with potential engagement in mitochondrial autophagy; SEMA4B may contribute to altered cell migration and apoptosis; RALGAPB implies regulatory involvement in cellular processes through GTP hydrolysis; EPHB1, as a tyrosine kinase, may signify engagement in signaling pathways crucial for cellular adaptation; SAR1A, EIF3M, and PWP2 may affect protein transport and synthesis, respectively; PHF5A may affect mRNA splicing; MFN2, being a multifunctional protein, has been examined in our subsequent investigations. These findings provide insights into the specific genes that exhibit significant changes in response to ischemic reperfusion injury.

As previously mentioned, the spliceosome has exhibited significant relevance both in biological processes and KEGG pathways. This suggests a potential close relationship between the spliceosome and MAMs, particularly in the context of ischemic reperfusion injury. However, there is currently limited research on the direct connection between the spliceosome and MAMs. Intriguingly, in a study of testis proteomics, the spliceosome also emerges as highly significant in the KEGG pathway, although the author does not delve extensively into this aspect. It's worth noting that a latest study has shed light on two splice variants of MFN2, namely ERMIN2 and ERMIT2, located on the endoplasmic reticulum (ER). These variants have been found to play crucial roles in maintaining communication between the ER and mitochondria, regulating endoplasmic reticulum morphology, facilitating calcium ion transport, and more [46]. This discovery could potentially mark the beginning of a new research avenue investigating the intricate relationship between splicing process and MAMs.

MAMs are intricately regulated by the actin cytoskeleton, which plays a pivotal role in driving fission and mitochondrial dynamics. The actin cytoskeleton emerges as a major driving force for the functional interplay between the mitochondria and ER, exerting its influence on diverse cellular aspects, including maintaining cellular morphology, regulating organelle distances, modulating signal transduction, and sustaining overall cellular function. Conversely, it is noteworthy that actin polymerization, a fundamental process in the actin cytoskeleton dynamics, is energy-demanding [47]. Significantly, our protein quantification analysis in the context of HR in HK-2 cells revealed a total of 25 actin or actin-related proteins. Among these, four genes—ACTR8, ARPC2, CAPZA2, and INF2—were found to be differentially expressed, with ACTR8 being upregulated and the remaining three proteins downregulated. The dynamic regulation of the actin cytoskeleton is a critical aspect of cellular responses to stress, including hypoxia/reoxygenation (H/R) injury. In our proteomic analysis of HK-2 cells subjected to H/R, we identified alterations in four key proteins associated with actin dynamics: ACTR8, ARPC2, CAPZA2, and INF2. ACTR8, an essential component of the Arp2/3 complex, showed increased expression, suggesting heightened actin branching. Concurrently, ARPC2, another Arp2/3 complex subunit, exhibited downregulation, potentially impacting actin nucleation [48]. CAPZA2, a subunit of the F-actin-capping protein, displayed decreased levels, indicating a potential reduction in the capping activity of F-actin [49]. INF2, a formin protein involved in actin polymerization, showed decreased expression, suggesting alterations in actin filament formation [50]. These dynamic changes in actin-related proteins likely contribute to the cellular responses associated with cytoskeletal remodeling, migration, and adaptation to the challenges imposed by hypoxic/reoxygenation stress.

Notably, MAMs play a crucial role in regulating calcium homeostasis, a factor that should not be overlooked. Consistent with previous studies, we also have identified those calcium channel proteins, such as IP3R, GRP75, VDAC1, SERCA, CANX, and so on. Under pathological conditions, including IRI, the normal maintenance of the number and function of MAMs may contribute to coordinating ATP production and timely clearance of damaged mitochondria through mitophagy, thus alleviating renal ischemic reperfusion injury. However, the abnormal and sustained increase of MAMs may also aggravate calcium overload and the transfer of oxygen free radicals, thus aggravating renal ischemic reperfusion injury. The changes of MAMs in different stages and degrees may play different roles. How to maintain a moderate number of MAMs and maintain their normal function is also a problem that needs to be further explored and solved. In this report, there wasn't significant difference between two groups. We speculate that there may be a time lag between the changes in mitochondrial dynamics and the occurrence of calcium imbalance in different stages of renal ischemic reperfusion. What we observed pertains to the initial phase of ischemic reperfusion, and the alterations in mitochondrial morphology and function may occur prior to the onset of calcium overload. The sequence of occurrence of various pathophysiological processes needs more in-depth research.

### Validation of BNIP3 and MFN2 and further exploration of the role of MFN2 in IRI

As previously mentioned, validation experiments for BNIP3 and MFN2 were conducted, and the results from both in vivo and in vitro experiments were consistent with the findings from the proteomics analysis. BNIP3 showed a significant upregulation, whereas MFN2 exhibited a significant downregulation. Both of them are located in the mitochondrial outer membrane. BNIP3 is believed to play a crucial role in ischemic reperfusion injury, primarily through the induction of mitophagy mediated by multiple pathways. HIF-1α-BNIP3-mediated mitophagy has been confirmed plays a protective role in IR of kidney, heart and cerebrum [51–53]. BNIP3, with its role in mitophagy, presents an intriguing avenue for therapeutic strategies aimed at clearing damaged mitochondria and alleviating injury. Therefore, the elevation of BNIP3 following HR may be a stress-induced self-protective mechanism in the HK-2 cells. Based on the aforementioned study, BNIP3 may represent a compelling target for therapeutic strategies focused on the clearance of damaged mitochondria and alleviation of injury.

MFN2, apart from being among the top differential proteins, also emerged as a hub gene according to bioinformatics analysis, emphasizing its central role. MFN2 is a multifunctional protein with the following primary functions: (i) As a GTPase, it plays a central role in mitochondrial metabolism; (ii) As a mitochondrial fusion protein, it participates in mitochondrial fusion and contributes to the maintenance and functionality of the mitochondrial network, including its regulation of mitochondria-associated ER membranes [54–56]; (iii) It is involved in the clearance of damaged mitochondria through the process of mitophagy [57]; (iv) It plays a role in controlling the unfolded protein response during endoplasmic reticulum (ER) stress [58, 59]. These diverse functions underscore the protective role of MFN2 in the IR injury. In our observations, we noted a significant reduction in MFN2 levels during ischemic reperfusion. Additionally, by using of two different techniques, confocal microscopy and transmission electron microscopy, we observed an increase in mitochondrial fission, mitochondrial swelling, and a perturbation in MAMs during IR. These phenomena may or at least partly be attributed to the decrease of MFN2. Furthermore, when MFN2 was overexpressed, we observed a reversal of the aforementioned mitochondrial damage and restoration of MAMs. Therefore, our results provide further confirmation of the protective role of MFN2 in the context of ischemic reperfusion injury.

To further explore the role of MFN2 in renal tubules and its underlying mechanisms and associated pathways, we conducted a proteomic study on normal HK-2 cells and OE-MFN2 HK-2 cells. KEGG enrichment analysis revealed a significant positive correlation between the PI3K pathway and MFN2. Subsequently, we performed a correlation analysis between upregulated genes in the PI3K/AKT pathway and MFN2. The genes identified in the PI3K/AKT pathway included LAMA5, ITGB3, PRKCA, PPP2R5C, OSMR, THBS1, and TLR2. LAMA5, ITGB3, PRKCA, and PPP2R5C have been implicated in various cellular processes, such as cell adhesion, cell transformation, and cell cycle checkpoint regulation. However, their specific roles in the context of IRI remain limitedly studied. OSMR, regulated by the PI3K/AKT pathway, has been shown to exert protective effects in acute kidney injury (AKI) [60]. THBS1 is rapidly upregulated in renal IRI, exacerbating kidney damage by stimulating ROS production and inducing apoptosis in renal tubular epithelial cells. Inhibition of THBS1 has been demonstrated to alleviate renal injury [61]. TLR2, a member of the Toll-like receptors (TLRs) family, has been extensively studied in renal IRI and is recognized as a crucial initiator of inflammatory responses leading to renal injury and dysfunction in IR injury. TLR2 activates multiple pathways, including MYD88/NFκB, MAPK/JNK, PI3K/AKT, contributing to the production of proinflammatory cytokines, chemokines, increased ROS accumulation, cell apoptosis, and exacerbation of graft rejection [62–64]. In conclusion, the findings shed light on the potential significance of these genes in the context of renal IRI. Understanding their specific roles and interactions may pave the way for targeted therapeutic strategies aimed at mitigating the impact of IRI on renal function.

Subsequently, we validated the PI3K/AKT pathway. Western blot results were consistent with the proteomic findings, showing that MFN2 overexpression upregulated p-PI3K and p-AKT. Generally, activation of the PI3K/AKT pathway is considered protective IRI across different organs such as the kidney [65], brain [66], and heart [67]. The activation of PI3K/AKT provides protection through the upregulation of antioxidant, anti-inflammatory, and autophagy activities, inhibiting mitochondrial dysfunction and cardiomyocyte apoptosis. Numerous studies indicate that PI3K/AKT activation has a protective effect against myocardial ischemia–reperfusion injury (MIRI) [62]. Additionally, non-coding RNAs have been implicated in the regulation of oxidative stress by modulating signaling pathways [10, 68]. The PI3K/AKT pathway is involved in Nrf2 activation, and its inhibition weakens Nrf2 transcriptional activity. AKT activation enhances adaptation to oxidative stress by activating Nrf2-related antioxidant signaling [69, 70]. In summary, apart from its protective role through the regulation of mitochondrial dynamics, MFN2 may exert its protective effects in ischemic reperfusion injury by activating the PI3K/AKT pathway. In summary, MFN2 may emerge as a potential target for preserving mitochondrial and MAMs integrity during renal IRI. Translating these findings into clinical practice involves exploring pharmacological or genetic interventions to modulate MFN2 expression or activity. Additionally, interventions targeting MAMs in general, considering their central role in cellular processes, could offer broader therapeutic strategies.

### Limitations

Firstly, in selecting the HK-2 cell line as a model for hypoxia/reoxygenation experiments, it is essential to acknowledge that ideal in vitro models that precisely mimic the complexity of proximal tubule cells are limited. It's important to emphasize that the use of HK-2 cells represents a technical compromise, and the findings should be interpreted with an awareness of the inherent limitations associated with this cellular model while HK-2 cells have been widely used in similar studies. We have incorporated additional in vivo experiments to strengthen the translational relevance of our findings. However, it is important to note that further in-depth in vivo proteomic studies are warranted for a more comprehensive understanding.

Secondly, based on the structural and component characteristics, MAMs are believed to be highly flexible and adaptable structures capable of recruiting various signaling molecules according to the different pathophysiological states. Ischemic reperfusion injury involves multiple pathological and physiological processes, and the primary mechanisms at may differ at different stages of injury. Our study focused on the initial phase of ischemic reperfusion injury, and as a result, it may not fully reflect the dynamic changes of MAMs at different injury stages. Adding more observation time points could potentially provide valuable insights into the relationship between MAMs and ischemic reperfusion injury more systematically and comprehensively.

### Potentially further and futuristic research

The current study paves the way for future research in several promising directions. One potential avenue is to delve deeper into the functional roles of specific proteins or pathways identified within MAMs and their contribution to renal ischemic reperfusion injury (IRI). By elucidating the precise mechanisms by which these proteins or pathways operate in MAMs, researchers can gain a better understanding of their involvement in the pathophysiology of renal IRI, potentially leading to the development of novel therapeutic strategies. Additionally, exploring potential therapeutic targets within MAMs to mitigate renal IRI and associated diseases represents an exciting prospect. By identifying and targeting specific molecules or pathways within MAMs that are dysregulated during renal IRI, researchers may be able to develop targeted interventions aimed at preserving mitochondrial function and mitigating tissue damage in the kidney. Exploring the application of emerging technologies, such as CRISPR-Cas9 or single-cell proteomics, could provide valuable insights into the intricate mechanisms underlying MAM dynamics during renal IRI. Moreover, our study hints at a potential link between spliceosomes and MAMs, suggesting an intriguing connection that warrants further investigation. Exploring the interplay between spliceosomes and MAMs could uncover novel regulatory mechanisms underlying mitochondrial dynamics and function in the context of renal IRI.

Overall, these future research directions have the potential to deepen our understanding of the molecular mechanisms underlying renal IRI and to uncover new therapeutic targets for the treatment of kidney injury and associated diseases. The identification of key proteins, pathways, and interactions within MAMs presents opportunities for developing targeted diagnostic and therapeutic strategies. These could include biomarkers indicative of MAM dysfunction or interventions aimed at modulating MAM-associated processes to ameliorate kidney injury. We have also underscored the need for further translational research to bridge the gap between laboratory findings and clinical applications. Future studies exploring the diagnostic potential of MAM-related biomarkers or testing interventions targeting MAMs in relevant clinical settings would be instrumental in realizing the clinical implications of our work.

## Conclusion

In conclusion, our investigation into the mitochondrial associated membranes (MAMs) proteomics of HK-2 cells subjected to hypoxia/reoxygenation (HR) revealed a pivotal role played by MAMs in various essential physiological and pathological processes. These include mitochondrial energy metabolism, mitophagy, endoplasmic reticulum stress cell division, apoptosis, and other critical cellular events. Notably, the key protein MFN2, located within MAMs, appears to exert significant protective effects during hypoxia/reoxygenation (HR) by preserving the stability and integrity of mitochondria and MAMs. These findings underscore the importance of MAMs and their associated proteins, particularly MFN2, in the intricate network of cellular responses to HR and provide valuable insights for further exploration into potential therapeutic strategies targeting MAMs in the context of renal IRI. Future studies should delve into specific aspects of MAM function and regulation, utilizing in-depth in vivo models to comprehensively unravel the dynamic changes occurring during different stages of renal IRI. In summary, our study elucidates the pivotal role of MAMs, notably MFN2, in mitigating renal ischemic reperfusion injury. This work enriches our understanding of cellular orchestration and acts as a compass for future research, inspiring innovative therapeutic strategies.

### Supplementary Information

**Additional file 1: Figure S1.** Schematic flowchart of the MAMs isolation and purification process. MAMs was isolated from HK-2 cells (N = 3, HR = 3) by applying with glass homogenization, differential centrifugations and a self-forming Percoll gradient centrifugation. Cytosol, pure mitochondria and ER were also isolated following the multiple centrifugation steps. Three replicates were used for each group.

**Additional file 2: Figure S2.** Western blot validation of MAMs purity. Cyto: cytoplasm, MAMs: mitochondrial associated membranes, PM: pure mitochondria, ER: endoplasmic reticulum. IP3R enriched in ER and low expressed in MAMs; MFN2 and GRP75 are as markers of MAMs and MFN2 also expressed in PM and ER; β-actin only expressed in Cyto; VDAC1 and COXIV are as markers of PM. TOM20 is a mitochondrial outer membrane protein which is mainly expressed in PM; CytC is a mitochondrial inner membrane protein which is only expressed in PM.

**Additional file 3: Table S1.** Subcellular distribution of total and differential proteins.

**Additional file 4: Table S2.** Enrichment analysis of total porteins.

**Additional file 5: Table S3.** Enrichment analysis of differential proteins.

**Additional file 6. Table S4.** The top 30 proteins in each of the five algorithms.

## Data Availability

The raw data used and/or analyzed during the current study are available from the corresponding author on reasonable request.
