# Supplementary figures and images for: Proteomic analysis of mitochondria associated membranes in renal ischemic reperfusion injury
Source: J Transl Med. 2024 Mar 10;22:261. doi: 10.1186/s12967-024-05021-0 (PMC10925013; doi:10.1186/s12967-024-05021-0)

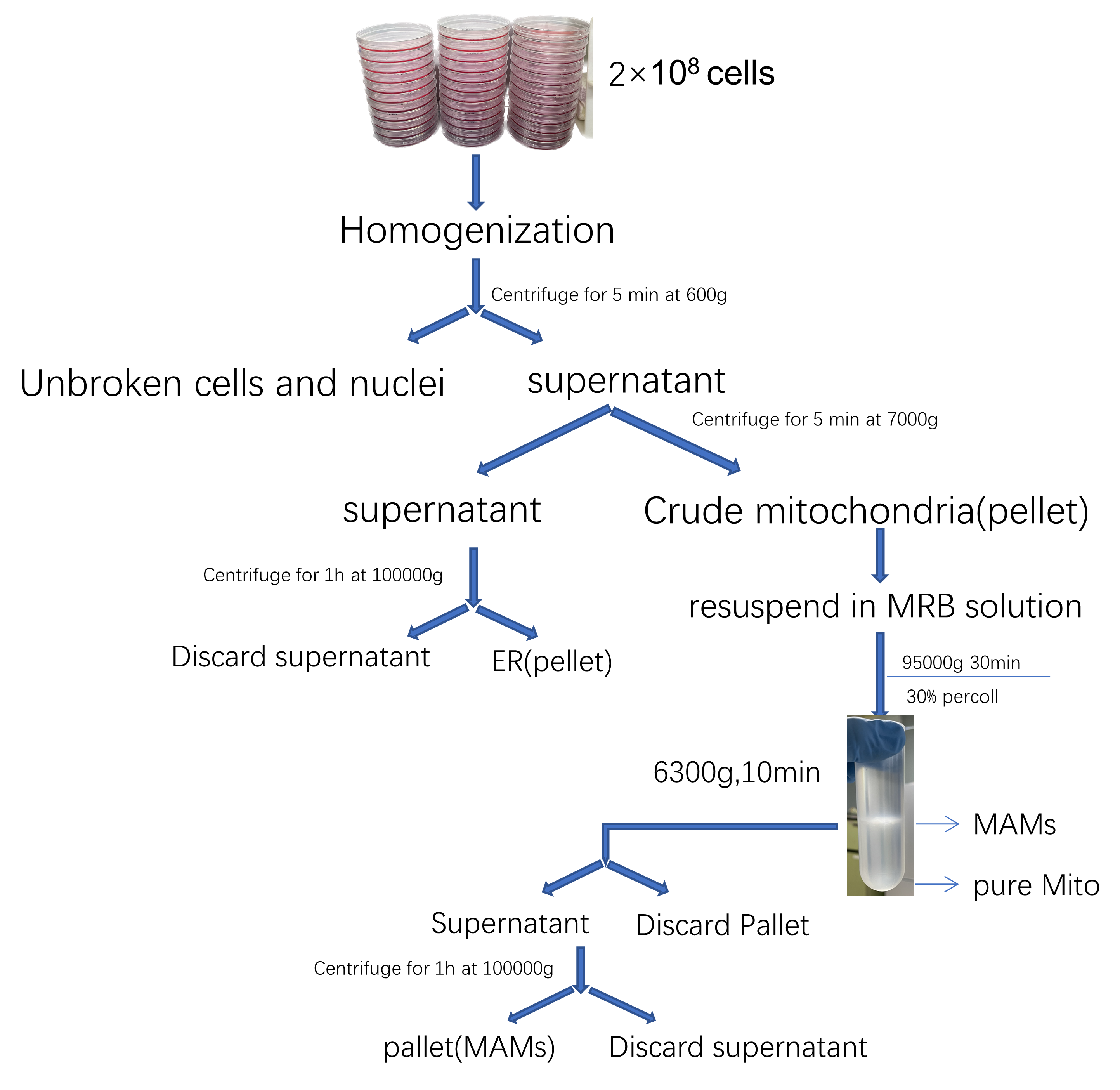

Supplement: Supplementary file 1 — Additional file 1: Figure S1. Schematic flowchart of the MAMs isolation and purification process. MAMs was isolated from HK-2 cells (N = 3, HR = 3) by applying with glass homogenization, differential centrifugations and a self-forming Percoll gradient centrifugation. Cytosol, pure mitochondria and ER were also isolated following the multiple centrifugation steps. Three replicates were used for each group. [file 12967_2024_5021_MOESM1_ESM.tif]

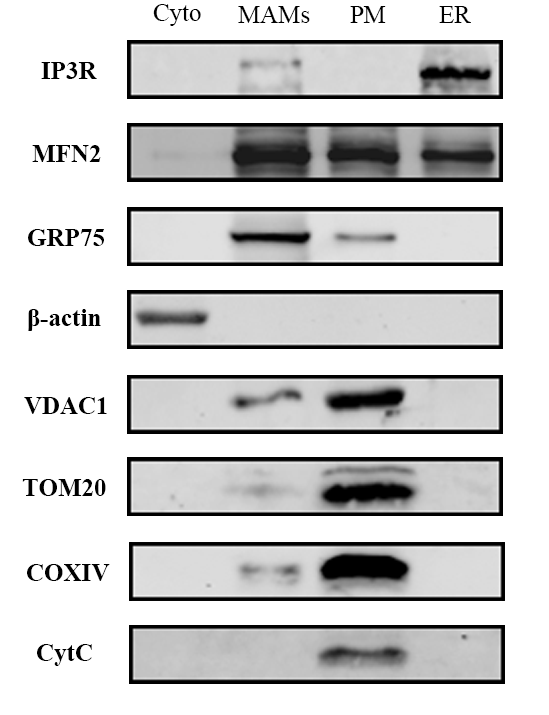

Supplement: Supplementary file 2 — Additional file 2: Figure S2. Western blot validation of MAMs purity. Cyto: cytoplasm, MAMs: mitochondrial associated membranes, PM: pure mitochondria, ER: endoplasmic reticulum. IP3R enriched in ER and low expressed in MAMs; MFN2 and GRP75 are as markers of MAMs and MFN2 also expressed in PM and ER; β-actin only expressed in Cyto; VDAC1 and COXIV are as markers of PM. TOM20 is a mitochondrial outer membrane protein which is mainly expressed in PM; CytC is a mitochondrial inner membrane protein which is only expressed in PM. [file 12967_2024_5021_MOESM2_ESM.tif]
